# Synergistic antitumor effect of a γ-secretase inhibitor PF-03084014 and sorafenib in hepatocellular carcinoma

**DOI:** 10.18632/oncotarget.26209

**Published:** 2018-10-09

**Authors:** Xuran Yang, Wei Xia, Lin Chen, Chuan Xing Wu, Cathy C. Zhang, Peter Olson, Xiao Qi Wang

**Affiliations:** ^1^ Department of Surgery, The University of Hong Kong, Hong Kong, China; ^2^ Oncology Research Unit, Pfizer Global Research and Development, La Jolla, California, USA

**Keywords:** GSI, PF-03084014, sorafenib, antitumor, HCC

## Abstract

As a multi-kinase inhibitor, sorafenib is beneficial in around 30% of hepatocellular carcinoma (HCC) patients; however, HCC patients develop acquired drug resistance quickly. Clinical benefits of sorafenib, in combination with transarterial chemoembolization (TACE), radiotherapy, and other chemodrugs are limited. We investigated the efficacy and mechanisms of Notch signaling inhibition as adjuvant to sorafenib in HCC spheroid-derived *in vitro* and *in vivo* tumor models, using the γ-secretase inhibitor (GSI), PF-03084014. The combination of PF-03084014 plus sorafenib inhibited proliferation and self-renewal of HCC spheroids (stem-like cancer cells). PF-03084014 significantly enhanced antitumor activity of sorafenib; both agents at low dose reached synergistic tumor growth suppression of HCC spheroid-derived orthotopic tumors. The Notch1-Snail1 signaling pathway contributed to sorafenib resistance via increasing epithelial-mesenchymal transition (EMT) and EMT-mediated cancer stem cell (CSC) features, such as increased expression of Snail1, N-cadherin, ABCG2, and the stem cell related genes Nanog and Oct4, and decreased expression of E-cadherin. Anti-tumor activity of the combination therapy was associated with decreased expression of survival signals (Mek/Erk, PI3K/Akt) and reduced microvessel density. PF-03084014 plus sorafenib targets Notch1-Snail1 signaling to reverse EMT and EMT-mediated CSC stemness in the tumors. These synergistic effects provide a rationale to utilize GSIs, in combination with sorafenib, as a new therapeutic strategy for the treatment of HCC.

## INTRODUCTION

Since approval by the FDA in 2006 for the treatment of advanced renal cell carcinoma, and in 2007 for the treatment of hepatocellular carcinoma (HCC), sorafenib is the only standard of care for advanced HCC patients. Sorafenib is an inhibitor of RAF serine/threonine kinases and receptor tyrosine kinases that are involved in VEGF1, 2, and 3, PDGFR, FLT3, c-Kit, and other signaling pathways such as STAT3. Thus, sorafenib inhibits the RAF-MEK-ERK/MAPK pathway to suppress tumor proliferation and is also a highly effective inhibitor of the pro-angiogenic VEGFs and PDGFR to suppress the microvasculature of tumors [1–4]. Moreover, sorafenib has been shown to induce apoptosis in tumors by several potential mechanisms that activate caspases [5, 6].

Clinically, sorafenib is beneficial in around 30% of HCC patients, although extended survival time is only 3–5 months. Considerable numbers of HCCs are refractory to sorafenib, as a result of primary or acquired resistance, which often develops within 6 months [7–9]. The genetic heterogeneity of HCC is responsible for primary resistance. Studies have shown that high activation of EGFR could be the determinant of primary resistance of HCC cells to sorafenib. Moreover, studies to identify predictive biomarkers of primary resistance have suggested that differential levels of pERK, JNK, and VEGFA might be the candidate markers for sorafenib response in HCC [3]. Alterations of several signaling pathways contribute to acquired sorafenib resistance, including the RAF/MEK/ERK and PI3K/Akt pathways, HGF/c-Met signaling, TGF-β signal-mediated epithelial-mesenchymal transition (EMT), hypoxia, and anti-apoptotic signaling pathways [10, 9]. Furthermore, emerging theories raise the prospects of immunomodulation on sorafenib sensitivity and capacity of HCC to hijack the existing vasculature in normal liver, limiting the need for angiogenesis and thereby providing resistance to the anti-angiogenic effects of sorafenib [10, 11, 4]. All of these studies indicate the multiplicity of driver genes and signaling pathways that limit the efficacy of sorafenib.

It has been observed that epithelial HCC cells are more susceptible to sorafenib, whereas HCC cells that have undergone EMT become not only invasive but also resistant to sorafenib. More importantly, these cells expressed the cancer stem cell (CSC) marker, CD44 [12, 9], which indicates a relationship between EMT, the emergence of CSCs, and drug resistance. The origin of CSCs in tumors is not fully understood. EMT induction in cancer cells results in the acquisition of CSC self-renewal capacity [13], which is a core contributor to tumor invasiveness, metastasis, therapy failure (caused by drug resistance), and recurrence [14]. Furthermore, recent studies indicate that the emergence of CSCs occurs partially as a result of EMT [13]. Thus, EMT-mediated CSC properties could be an important molecular mechanism of sorafenib resistance.

Targeting both EMT pathways and CSC maintenance is an attractive therapeutic strategy. However, EMT-based pharmacological strategies that directly target EMT-associated genes such as E-cadherin, N-cadherin, and vimentin are often ineffective [15, 16]. CSCs have been demonstrated to have one or more aberrations in various signaling pathways, including Notch, Hedgehog (HH), and Wnt, that control self-renewal and are important for embryonic developmental processes such as EMT, MET, and differentiation [17]. New agents targeting these “embryonic pathways”, in order to interfere with CSC maintenance, could be an effective option for overcoming sorafenib resistance.

PF-03084014 is a γ-secretase inhibitor (GSI) that has been shown to have antitumor effects on multiple tumors, including HCC [18–20]. In the present study we evaluated the antitumor efficacy of PF-03084014, a γ-secretase inhibitor (GSI), and sorafenib, individually and in combination, in an HCC spheroid-derived orthotopic model.

## RESULTS

### Low dose of PF-03084014 sensitized HCC spheroids to sorafenib *in vitro*

**Figure 1:**
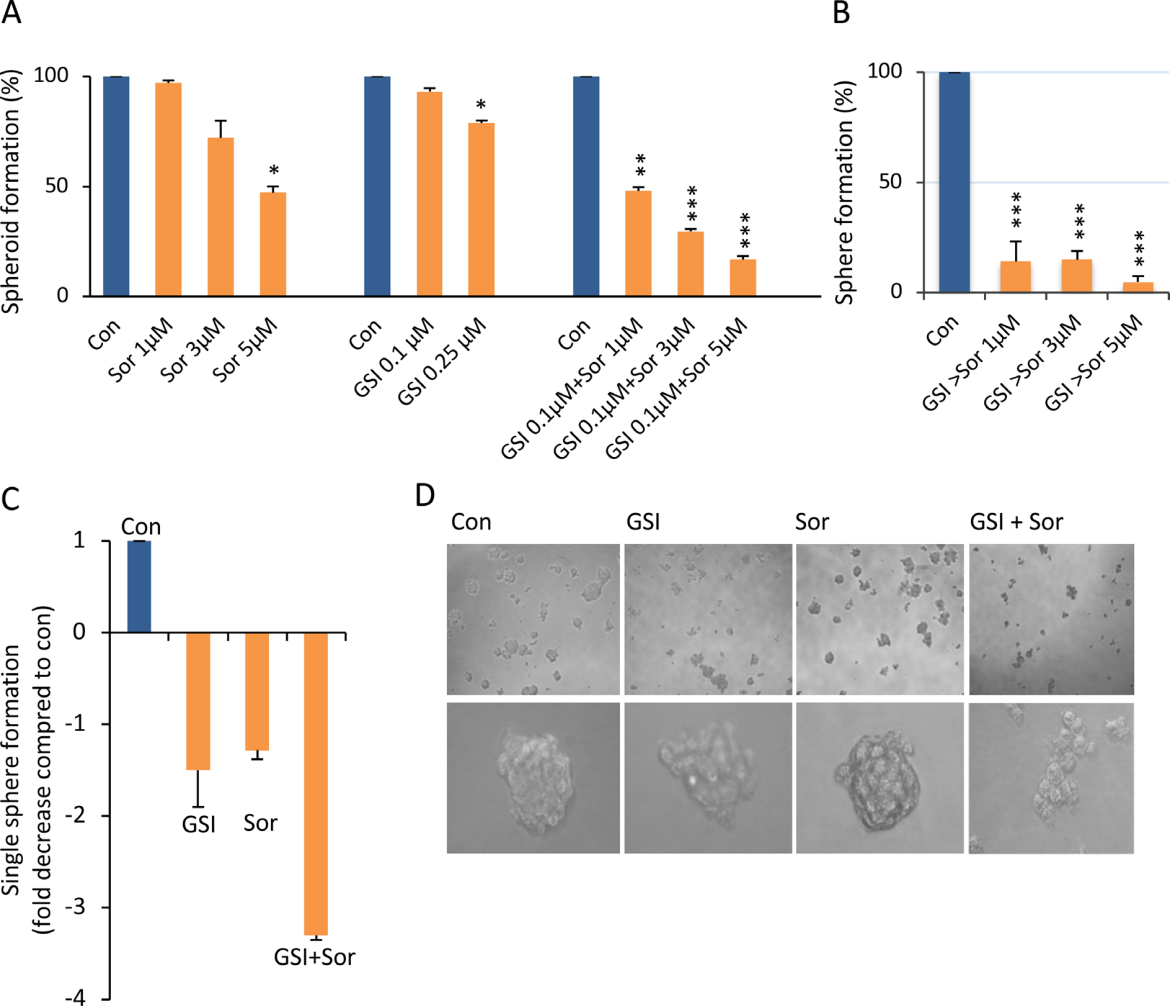
Sorafenib, in combination with PF-03084014, significantly increased the inhibition of HCC spheroid self-renewal and proliferation (**A**) HCC 97H-derived spheroid CSCs were treated with vehicle (DMSO) and sorafenib (sor, 1, 3, 5 μM (left panel) or PF-03084014 (0.1, 0.25 μM) (middle panel) alone, or PF-03084014 0.1 μM in combination with sorafenib 1, 3, and 5 μM, respectively (right panel). Overall spheroid formation (treatment vs. con) was calculated as a percentile. The data are presented as the mean ± SD, *n* = 3 from three independent experiments, each in triplicate. An independent *t* test was used for statistical comparison. ^*^p < 0.05; ^**^p < 0.01; ^***^p < 0.001. A synergistic effect was considered as the inhibition effect by PF-03084014 + sorafenib was greater than the sum of the effect by PF-03084014 and sorafenib alone. (**B**) 97H spheroids were pre-treated with PF-03084014 for 24 hrs followed by the addition of sorafenib (GSI > sorafenib). (**C**) The single spheroid formation capacity in the control was defined as 1, and the fold decrease in the PF-03084014 (0.1 μM) sorafenib (1 μM), or PF-03084014 + sorafenib groups were calculated as the inverse of the fold change. (**D**) Phase contrast images of spheroid colonies after treatment with DMSO or PF-03084014 and sorafenib alone, or PF-03084014 in combination with sorafenib.

Using liver cancer anchorage-independent spheroids as our model, which contain enriched CSCs [21], we found that MHCC97H (97H) HCC-derived spheroids were resistant to sorafenib, with suppression rate was 3% and 28% at 1 and 3 μM; only the higher dose of 5 μM of sorafenib could suppress HCC spheroid cell proliferation (suppression rate 53%) (Figure 1A, left panel). Notch signaling has great relevance to multiple aspects of cancer biology, such as CSC self-renewal, angiogenesis, and cancer immunity [17]. Increased expression of the active form of Notch1 was detected in 97L and 97H cells but not in other HCC cells in our previous study [22], leading to a hypothesis that sorafenib resistance might be modulated by Notch1. We therefore investigated whether the Notch inhibitor PF-03084014 could modulate sorafenib sensitivities. Low dose of PF-03084014 (0.1 μM) alone had no impact on HCC spheroid proliferation, and a higher dose of 0.25 μM had only a moderate effect, with inhibition rate was only 7% and 21% (Figure 1A, middle panel). However, low dose of PF-03084014 (0.1 μM) enhanced responsiveness to sorafenib by inhibition rate reaching 52%, 70%, and 83%, respectively (Figure 1A, right panel). The data indicates a synergistic effect as the inhibition effect by PF-03084014 + sorafenib was greater than the sum of the effect by PF-03084014 and sorafenib alone. If spheroids were pre-treated with the low dose of PF-03084014, followed by sorafenib treatment, spheroid proliferation was further suppressed, with the lowest survival rate of < 5% in 97H-spheroids (Figure 1B). Single cell-derived spheroid formation reflects CSC self-renewal capacity [17]. We then tested low doses of both PF-03084014 (0.1 μM) and serafenib (1 μM) in a single cell spheroid formation assay. Compared to control, PF-03084014 and sorafenib alone reduced single spheroid formation 1.5 and 1.28 fold, respectively, whereas the combination of the two drugs resulted in a 3.3 fold reduction in single spheroid formation (Figure 1C). The results indicate that the combination therapy of PF-03084014 plus sorafenib has enhanced suppression effects on HCC spheroid formation and self-renewal, indicating a synergistic effect (Figure 1A–1D).

### Combination of low doses of PF-03084014 and sorafenib enhanced antitumor effects synergistically in HCC spheroid-derived orthotopic tumors

**Figure 2:**
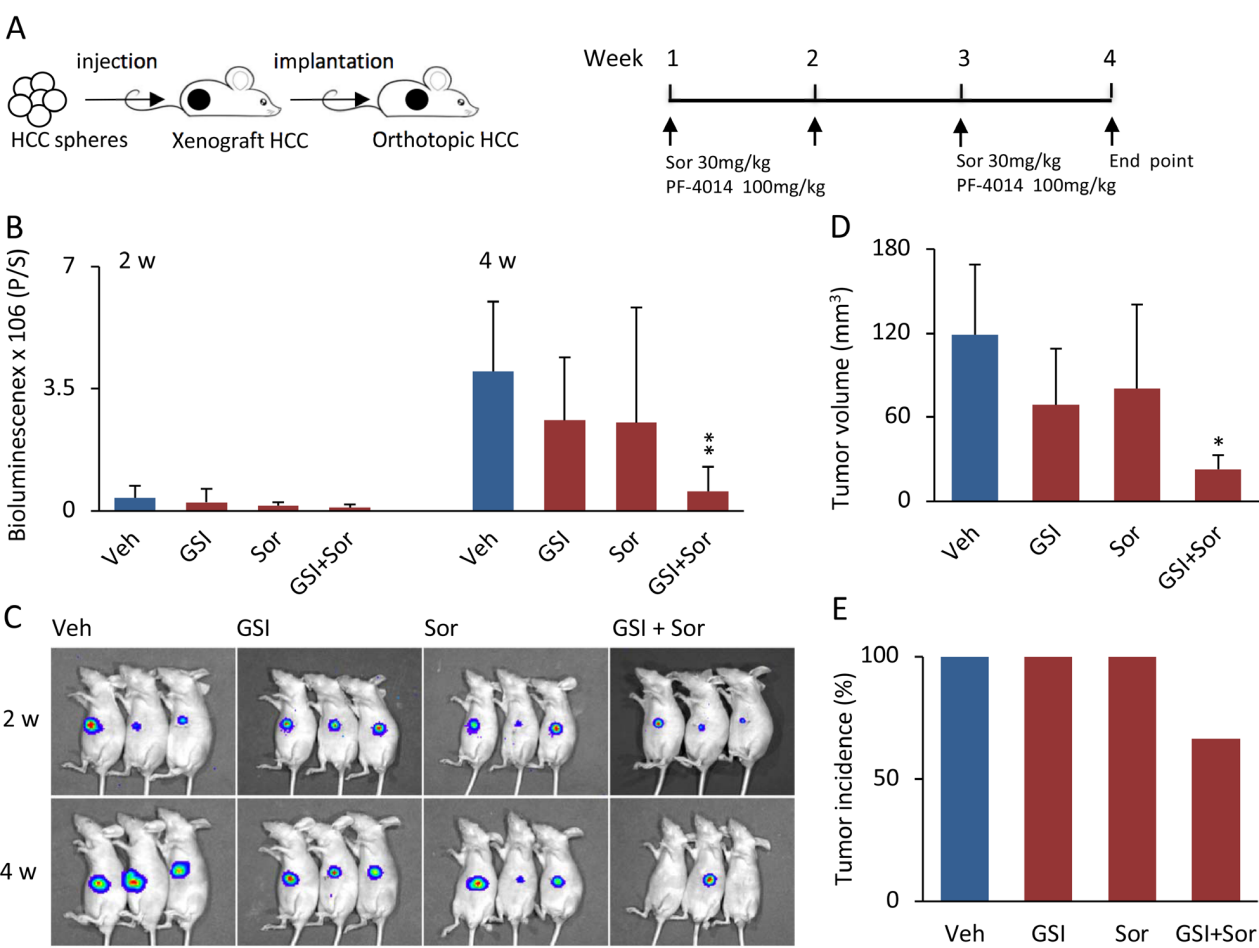
The combination of PF-03084014 with sorafenib displayed greater antitumor effects than either drug alone in the HCC spheroid-derived orthotopic model (**A**) Schema of the experimental setup. The 97H spheroid-generated subcutaneous tumor cubes were implanted into the left liver lobes of nude mice. One week after tumor implantation, the mice were randomized and treated orally with vehicle, PF-03084014, sorafenib, and PF-03084014 + sorafenib. (**B**) Tumor growth, based upon the luciferin bioluminescence signal, at 2 and 4 weeks. Data are presented as the mean ± SD, mouse number = 7 in each group. ^**^p < 0.01. (**C**) Representative tumor bioluminescence images at 2 and 4 weeks in vehicle, PF-03084014, sorafenib, and PF-03084014 + sorafenib, respectively. (**D**) Statistical comparison of the tumor volumes measured at the end point of the study (4 weeks). ^*^p < 0.05. (**E**) Orthotopic tumor incidence (%) of the respective treatment groups at the end point.

To determine whether the synergistic impact of the combination of PF-03084014 with sorafenib on HCC spheroid formation *in vitro* can be extended to *in vivo* models, orthotopic HCC tumors were generated from 97H spheroid-derived CSCs (Figure 2A, left panel), and treated with vehicle, PF-03084014 alone, sorafenib alone, and PF-03084014 plus sorafenib (Figure 2A, right panel). Both reagents, either alone or in combination, were administrated in low doses compared to the dosages previously applied in an *in vivo* HCC model [20, 23, 24]. Using bioluminescence to trace the tumor growth, we found that PF-03084014 alone (100 mg/kg/day) decreased tumor growth by 35% and sorafenib alone (30 mg/kg/day) decreased tumor growth by 37.5%, respectively. Compared to the vehicle group, tumor growth by drug alone did not reach statistical significance (Figure 2B–2D). However, the combination of the 2 agents in the same low doses increased antitumor efficacy dramatically, with tumor growth decreased by 85.85% (Figure 2B–2D). The decreased tumor effect of the combination treatment was greater than the sum of the inhibitory effects by PF-03084014 or sorafenib alone, indicating a synergistic impact. Moreover, tumor incidence in the combined treatment group was 66.7%, whereas it was 100% in vehicle, and PF-03084014 or sorafenib alone (Figure 2E). Both PF-03084014 and sorafenib have gastrolintestinal toxicity [25, 2]. In the present study, mouse body weights were not impacted by treatment (Supplementary Figure 1), suggesting that the administration strategy with low dosages and a 7-days-on/7-days-off schedule limited toxicity while reaching significant tumor inhibitory responses (Figure 2).

### Combination therapy inhibited tumor proliferation and blocked tumor angiogenesis *in vivo*

**Figure 3:**
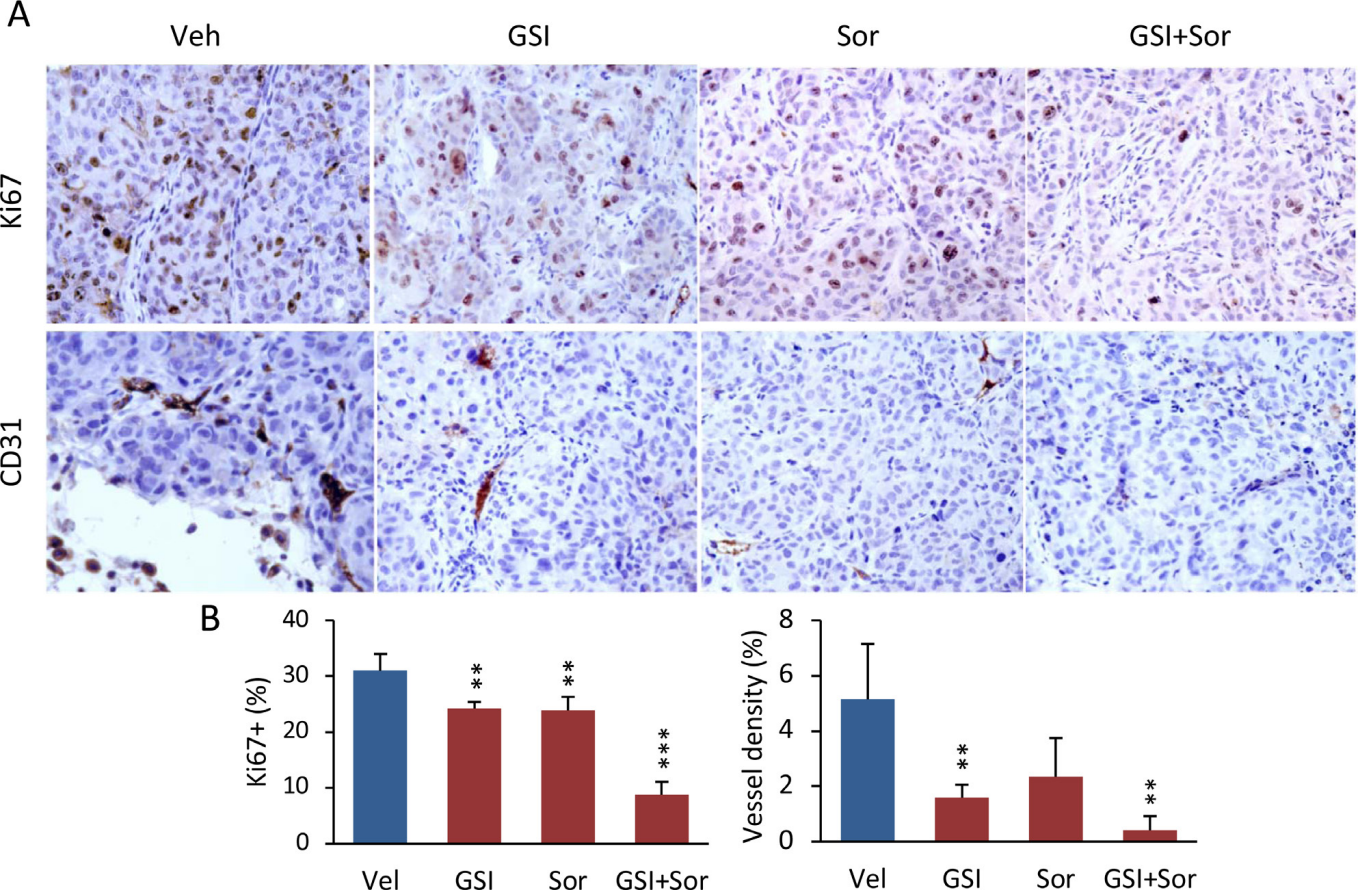
The combination of PF-03084014 with sorafenib inhibited tumor cell proliferation and tumor angiogenesis in mice (**A**) At the endpoint of the study (4 weeks after drug administration), tumors were harvested as described in Materials and Methods. A portion of the tumor section from each treatment group (*n* = 3, from different mouse tumor) was subjected to immunohistochemistry for measurement of proliferation (anti-Ki-67) and tumor angiogenesis (anti-CD31). Representative photomicrographs of Ki-67 and CD31-stained sections are shown. (**B**) Quantitation of Ki-67 (left) and CD31-positive percentages (right). ^**^p < 0.01; ^***^p < 0.001.

To understand the cellular mechanism that could contribute to the antitumor effects, we measured tumor proliferation by using Ki-67 expression and tumor vascularity, using anti-CD31 antibodies to identify microvessels. One of the primary antitumor effects is inhibition of VEGFs and PDGFR to suppress tumor angiogenesis [1–4]. Compared to the vehicle group, administration of PF-03084014 alone decreased Ki-67 expression by 22% and microvessel density by 69%; administration of sorafenib alone decreased Ki-67 expression by 23% and microvessel density by 54.4% in HCC-spheroid tumors. However, the combination of both drugs reduced Ki-67 expression by 71.6% and microvessel density by 91.8% (Figure 3A, 3B). The reduced levels of Ki-67 expression and microvessel density were more than 2 fold in the combination group compared to either PF-03084014 or sorafenib alone. These results indicated that the combination therapy, even at low doses for each agent, had a greater impact on tumor proliferation than either agent alone, thereby reaching a level of synergistic inhibition.

### Notch1-Snail1 mediated EMT in sorafenib resistance

**Figure 4:**
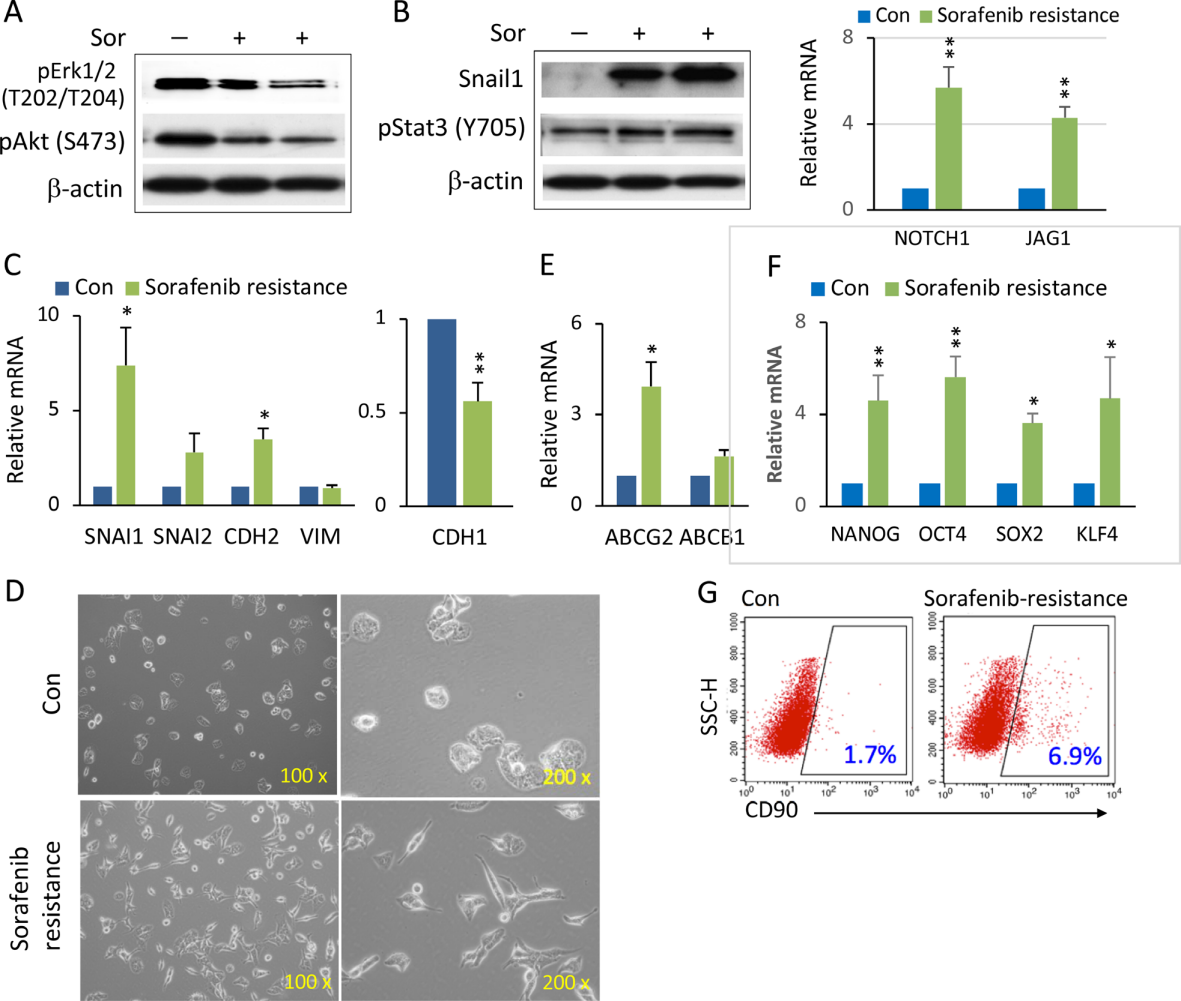
Enhanced Notch1 and Snail1 expression and EMT-mediated stemness in sorafenib resistant HCC spheroids (**A**) 97H spheroids were treated with high doses of sorafenib (10–15 μM) for over two weeks to generate sorafenib resistant cells. Western blot analysis of phospho-Erk1/2 and phospho-Akt in sorafenib-resistant (Sor+) cells compared with control (Sor−). (**B**) Western blot analysis of Snail1 and pStat3 in sorafenib-resistant (Sor+) cells compared with control (Sor−) (left panel). mRNA levels of NOTCH1 and its ligands JAG1 in sorafenib-resistant spheroids versus control (right panel). (**C**) mRNA levels of the EMT related genes SNAIL1, SNAIL2, CDH2 (N-CADHERIN), VIM (VIMENTIN) (left panel), and CDH1 (E-CADHERIN) (right panel) in sorafenib-resistant 97H spheroids compared to control (non-sorafenib resistance). (**D**) Phase contrast images of the cell morphologies of sorafenib-resistant cells compared with control. (**E**) mRNA levels of the multidrug resistant genes, ABCG2 and ABCB1, in sorafenib-resistant 97H spheroids versus control. (**F**) mRNA levels of the stemness genes, NANOG, OCT4, SOX2, and KLF4, in sorafenib-resistant and control spheroids. qPCR data are represented as the mean ± SD, *n* = 2 (from different sorafenib-resistant populations). An independent *t* test was used for statistical comparison. ^*^p < 0.05; ^**^p < 0.01. (**G**) Flow cytometry analysis of the CD90+ populations, shown by dot blot, in sorafenib-resistant versus control cells.

We wished now to explore the underlying molecular mechanism of the synergistic effects of combined Notch inhibitor and sorafenib, and whether modulating the Notch receptor resulted in sensitization of HCC spheroids to sorafenib. To do this we generated and investigated the sorafenib-resistant 97H spheroids and 97L cells. Conventional sorafenib-resistant pathways, such as phosphorylation of Erk and Akt, were downregulated in sorafenib-resistant 97H spheroids (Figure 4A), indicating that the RAF/MEK/ERK and PI3K/Akt pathways were not responsible for the acquired sorafenib resistance. Snail1 expression was dramatically enhanced in sorafenib-resistant spheroids (Figure 4B, left panel). mRNA levels of other EMT genes such as CDH2 (N-cadherin) were increased, whereas CDH1 (E-cadherin) was decreased (Figure 4C). More importantly, the levels of NOTCH1 and its ligand JAG1 were significantly enhanced in sorafenib resistant 97H spheroids (Figure 4B, right panel). It is possible that this may be a key player in sorafenib resistance via Notch-mediated EMT. Aberrant Notch1 activation has been shown to be a predictor of poor prognosis in HCC patients due to Notch1-Snail1 signaling associated with tumor metastasis [22]. In a larger data set from 423 of liver cancer patients (The Cancer Genome Atlas-Cancer Genome, TCGA liver cancer), both NOTCH1 and SNAIL1 showed a significant expression correlation (Supplementary Figure 2), suggesting a potential for the two proteins functioning together. The phosphorylation of Stat3, which is another activator of EMT [26], was also increased (Figure 4B, left panel). Sorafenib resistant 97L monolayer cells also changed in appearance, with many cells acquiring a mesenchymal-like shape (Figure 4D, lower panel). The indication is that sorafenib-resistant cells underwent EMT via enhanced Notch1-Snail1 signaling (Figure 4B–4D). Thus, the Notch1-Snail1 signaling pathway activation not only played a role in HCC tumor pathogenesis, but also contributed to sorafenib resistance. Furthermore, expression of the drug transporter gene, ABCG2, but not ABCB1, was also enhanced in sorafenib resistant cells (Figure 4E). As a multi-drug resistance gene, enhanced ABCG2 expression also might have a role in increasing the level of sorafenib resistance. It has been recognized that induction of EMT in cancer cells is accompanied by the acquisition of CSC properties, such as self-renewal and drug resistance. We found enhanced expression of the stem cell - associated genes, NANOG, OCT4, and KLF4, in sorafenib resistant HCC spheroids (Figure 4F). The liver CSC marker, CD90, was also enriched in sorafenib resistant 97H monolayer cells (Figure 4G).

### Knockdown of SNAIL1 increased sorafenib sensitivity via reversed EMT

**Figure 5:**
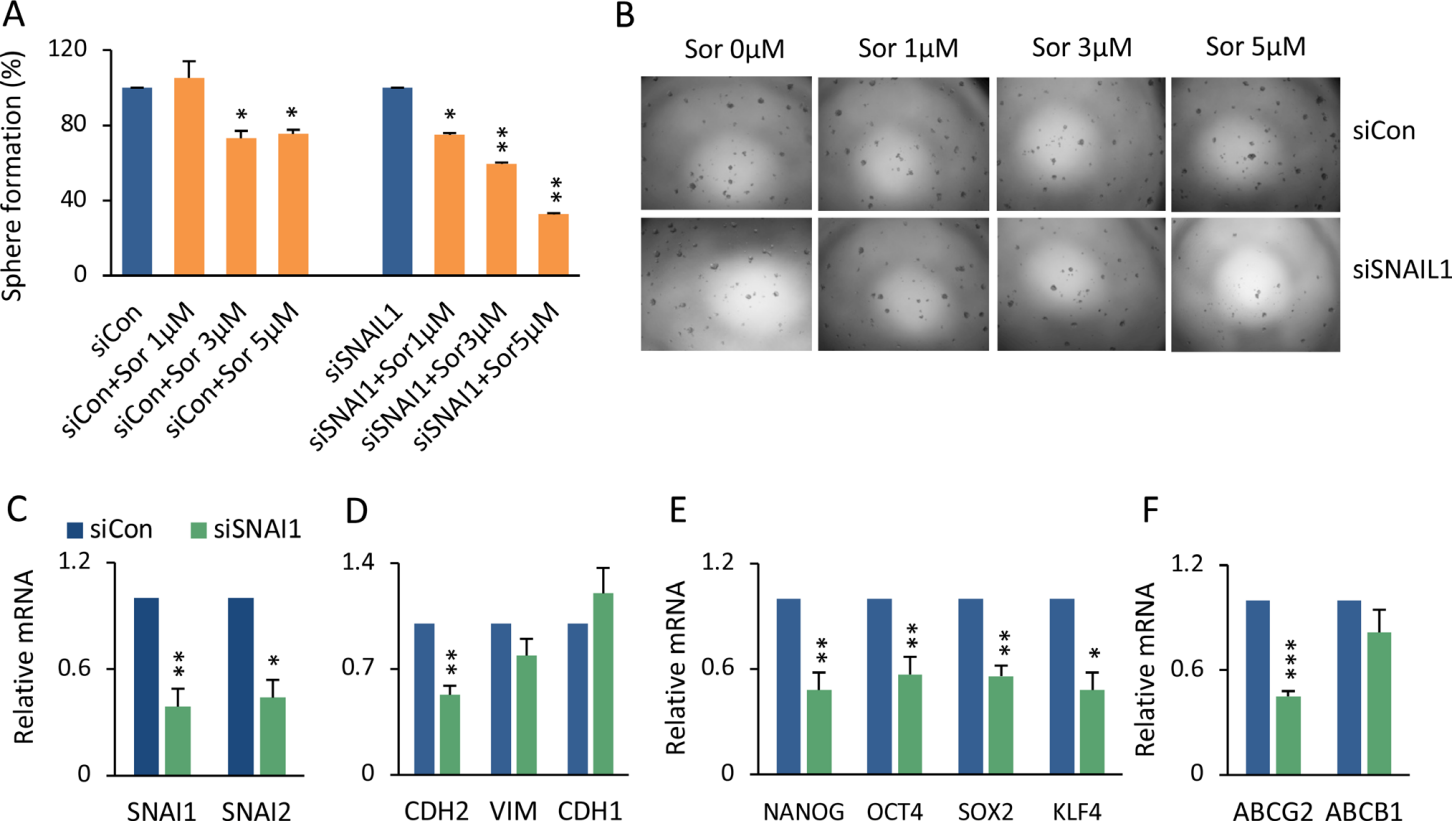
Genetic knockdown of SNAIL1 sensitized HCC spheroids to sorafenib (**A**) 97L monolayer cells were transfected with siRNA-SNAI1 or siRNA-control, followed by sorafenib 1, 3, or 5 μM treatment for 48 hrs. The cells were then seeded for spheroid formation. The percentage of formed spheroids was calculated as sorafenib versus vehicle group in both siRNA-SNAI1 and siRNA-Control cell cultures. The mean ± SD was from two independent siRNA transfection experiments, ^*^p < 0.05; ^**^p < 0.01. (**B**) Phase contrast images of sphere colonies of (A). (**C**–**F**) Statistical comparison of mRNA levels of SNAIL1, SNAIL2 (C), CDH2, VIM, CDH1 (D), NANOG, OCT4, SOX2, KLF4 (E), and ABCG2 and ABCB1 (F) between siRNA-SNAI1 and siRNA-Control, ^***^p < 0.001.

In the sorafenib resistant cells, whereas not all EMT components were altered, elevated Snail1 expression dramatically stood out (Figure 4B, 4C). The exact mechanistic correlation between EMT and sorafenib resistance is still unknown, and it is uncertain whether EMT is a trigger or the result [4]. We attempted to distinguish these possibilities by knockdown of SNAIL1 expression, using small interfering RNA (siRNA). When treated with siRNA-SNAI1, a low dose of sorafenib significantly decreased overall spheroid formation compared to control siRNA (Figure 5A, 5B). siRNA-SNAIL1 not only knocked down expression of SNAIL1 but also SNAIL2 and CDH2, significantly (Figure 5C, 5D), indicating that Snail1 might be able to modulate other EMT genes. This hypothesis was supported by the finding that expression levels of SNAIL1 were significantly correlated with VIM (Vimentin) and TWIST1 in 423 liver cancer patients (Supplementary Figure 3) (TCGA liver cancer). Interestingly, by knocking down SNAIL1, expression of the stemness-related genes, NANOG, OCT4, SOX2, and KLF4 (Figure 5E), as well as the multiple drug resistant gene ABCG2 were all decreased (Figure 5F).

### Combination therapy reduced EMT and CSC stemness

**Figure 6:**
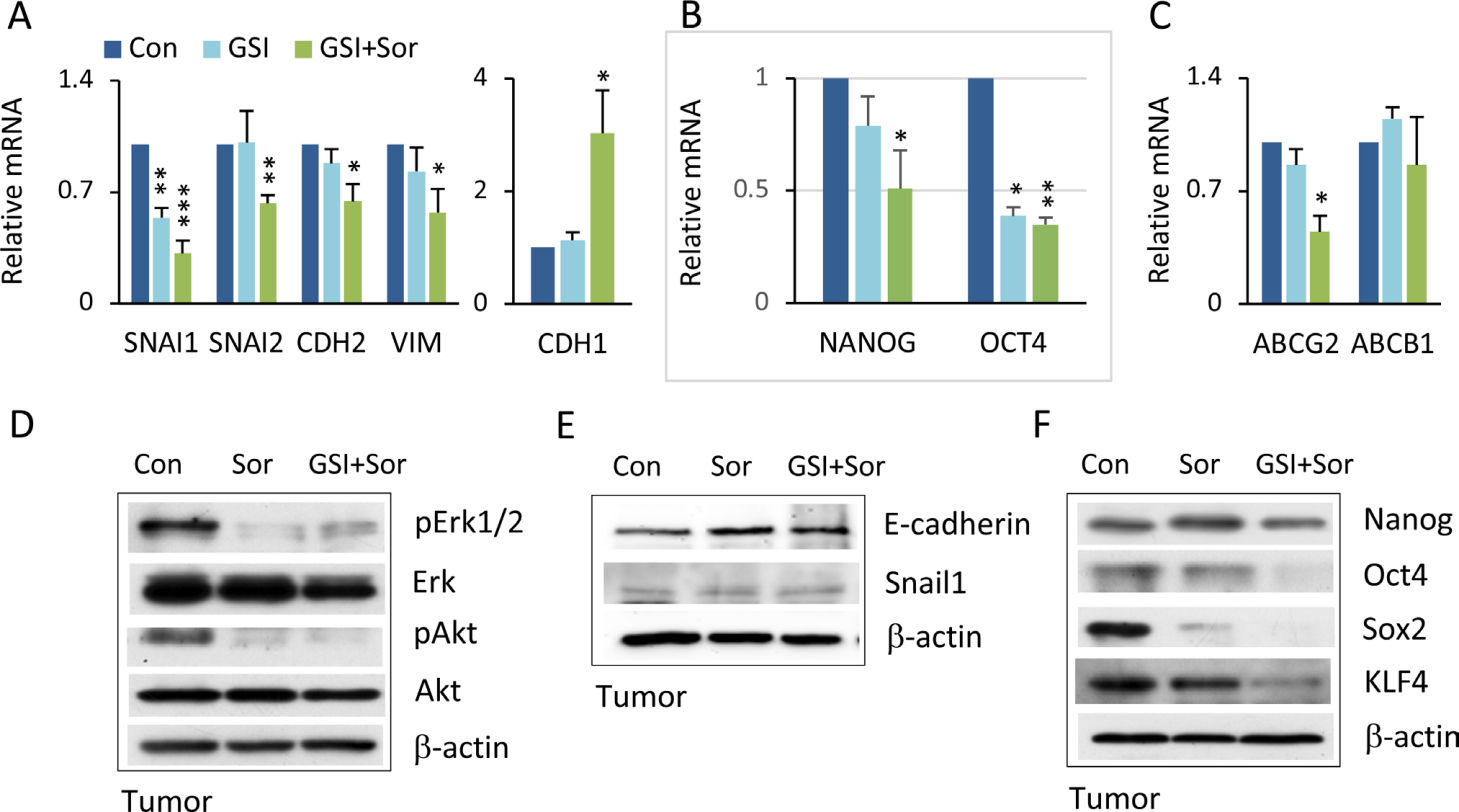
Combination therapy inverted EMT and CSC stemness (**A**) HCC spheroids treated with vehicle, PF-03084014, sorafenib, and PF-03084014 + sorafenib for 48–72 hrs followed by qRT-PCR analysis. The statistical comparison is of mRNA levels of SNAIL1, SNAIL2, CDH2, VIMENTIN, and CDH1 in drug treated cells versus control. (**B**) mRNA levels of stemness genes NANOG and OCT4 in drug treated cells versus control. (**C**) mRNA levels of ABCG2 and ABCB1. qPCR data are the mean ± SD, *n* = 2. ^*^p < 0.05; ^**^p < 0.01; ^***^p < 0.001. (**D**) At the end of study, tumors were harvested from mice as described in Materials and Methods. Proteins were extracted and subjected to immunoblot analysis for phospho-Erk and phospho-Akt. (**E**) Immunoblot analysis for E-cadherin and Snail1. (**F**) Immunoblot for stemness proteins.

We next investigated whether the combined actions of PF-03084014 and sorafenib, which showed a synergistic impact on inhibition of tumor growth (Figure 2), could reverse the EMT phenotype as well as EMT-mediated CSC stemness. *In vitro*, the combination of PF-03084014 and sorafenib at low doses reversed EMT related gene expression, with the most significant effect on reduction of SNAIL1 (Figure 6A). It noted that CSC-associated gene expression of NANOG and OCT4, as well as ABCG2, were significantly decreased (Figure 6B, 6C). In tumor tissues, similar effects were also observed, such as reduced activation of Erk and Akt (Figure 6D), reversed Snail and E-cadherin expression (Figure 6E), and down-regulated stemness-associated protein expression (Figure 6F). It noted the differences of EMT and CSC associated gene expression between regulating by siRNA-SNAIL1 and by drug treatment, likely due to the combined GSI and sorafenib treatment could have vary mechanisms (Figures 5C–5F, 6A–6C).

## DISCUSSION

In this study we demonstrated that applying a GSI (PF-03084014) in combination with sorafenib had anti-tumor effects in an HCC spheroid-derived orthotopic tumor model. Both agents alone at low dose did not have a significant inhibitory effect, whereas the combination of GSI and sorafenib (both at the same low dose) had a significant inhibitory effect, reaching a synergistic impact. We found that anti-tumor activity of the combined therapy was associated with inhibition of both proliferation and angiogenesis. Furthermore, GSI-mediated anti-tumor effects resulted in targeting Notch1- and Snail1-mediated EMT and CSC stemness. These results provide evidence: (a) that Notch inhibitor might have therapeutic benefits via enhancing efficacy of sorafenib for HCC patients; and (b) that GSI and sorafenib, in targeting the Notch1-Snail1 pathway, provides a potential molecular mechanism.

Sorafenib, as a multi-kinase inhibitor, remains the only approved systemic treatment for advanced HCC, although the therapeutic benefits are modest due to intrinsic and acquired drug resistance [2]. There have been many preclinical research and clinical trials on sorafenib combination therapy, including TACE (transarterial chemoembolization), radiotherapy, and the chemotherapeutic drugs doxorubicin and erlotinib [27]. However, clinical benefits from these combination therapies have not significantly improved survival [27]. Our study is the first to combine the Notch inhibitor, GSI, with sorafenib in an HCC spheroid-derived orthotopic model. Our strategy of combining GSI with sorafenib resulted in synergistic impact in inhibition of both HCC spheroid cultures (*in vitro*) and orthotopic tumor growth (*in vivo*), whereas the same low dose of either agent alone had no significant effect. Successful combinatorial treatments may produce additive or synergistic effects, where the degree of synergy and benefit is the more critical determinant [28]. Both GSI and sorafenib produce gastrointestinal toxicity. Lowering the dose of both drugs in combination reduces this toxicity but the therapeutic effect is retained.

Sorafenib mediates its antitumor activity via several mechanisms, including targeting: (i) the RAF-MEK-ERK/MAPK pathway to inhibit proliferation; and (ii) VEGFs and PDGFR to inhibit angiogenesis [1–4]. Sorafenib resistance has been associated with alterations in these pathways [9, 10]. Our study demonstrates that the Notch1-Snail1 pathway is also responsible for sorafenib resistance. We found high expression of Snail1 in sorafenib resistant HCC cells, which was mediated by activated Notch1. Snail1 also regulated other EMT genes and EMT-related CSC features. Furthermore, down-regulation of Snail1 by siRNA knockdown, coupled with administration of GSI, led to sorafenib sensitization. Previously, suppression of Notch1 activity was shown to result in effective reduction of EMT *in vitro* and tumor metastasis *in vivo* [20, 22]. Thus, GSI-mediated inhibition of the Notch1-Snail1 pathway is a reasonable choice for combination with sorafenib, in order to overcome resistance. Indeed, as our results show, GSI + sorafenib induced more than two fold efficacy than either single agent alone. Similar to sorafenib, the GSI, PF-03084014, also inhibited Erk, Akt and Stat3 activity [20]. Thus, PF-03084014 + sorafenib has the potential to simultaneously affect multiple cell types within a tumor, inducing enhanced effects on anti-proliferation and anti-angiogenesis. Notch1-Snail1 pathway is identified for sorafenib treatment failure in HCC in this experiment setting, although other mechanism could not be excluded in the complex of sorafenib resistance.

GSIs have been demonstrated to have anti-CSC activity in breast cancer [17, 18, 29, 30]. Our observation that sorafenib alone resulted in an increased population of liver CSCs, and the associated expression of stem cell related genes is consistent with the chemoresistant nature of CSCs, which, in this study, likely resulted from Notch1-Snail1 mediated EMT. GSI sensitized HCC spheroids and tumors to sorafenib, and was associated with other phenotypical changes. These included: decreased Snail1 expression and reversing altered EMT gene expression; impaired stem cell associated gene and protein expression (Nanog, Oct4, Sox2, and KLF4); and decreased expression of the drug resistance gene, ABCG2. Moreover, SNAIL1 knockdown generated a similar set of effects.

In summary, Notch1-Snail1 signaling pathways are not only associated with late stage and metastatic HCC disease [22] but, as these studies document, also contribute to HCC sorafenib resistance. Our previous study demonstrated inhibitory effects of GSI (PF-03084014) in the HCC model, but this effect was seen only at relatively high doses of GSI [20]. In the same HCC model, treatment with a combination of PF-03084014 and sorafenib resulted in statistically significant tumor suppression compared to either agent alone. Importantly, both PF-03084014 and sorafenib were needed only at a low dose to reach a synergistic effect. Thus, this combined therapy of GSI with sorafenib suggests a promising new strategy for the treatment of HCC.

## MATERIALS AND METHODS

### Liver cancer spheroid assay and drug treatment

The HCC lines, MHCC97H (97H) and MHCC97L (97L), were cultured in DMEM supplemented with 10% FBS (Life Technologies) at 37° C and 5% CO_2._ 97H and 97L cells were isolated from a male metastatic HCC patient [31] and transfected with luciferase. The generation of liver cancer spheroids from 97H and 97L followed the method described previously [32]; in brief, cancer spheroids were cultured in DMEM:F12 (Life Technologies) supplemented with 2% B-27 (Life Technologies), EGF, bFGF (PeproTech), 100 IU/ml penicillin, and 100 μg/ml streptomycin on ultra-low attachment plates for 10–12 days. The numbers of cells in spheroid colonies larger than 50 μM were counted [33]. For single cell formation capacity, HCC spheroids were dissociated with TrypLE and serially diluted for single spheroid formation. The spheroids for all of the experiments were derived from the expansion from the second generation of a single spheroid colony. Sorafenib (Selleck Chemicals) (1–5 μM), γ-secretase inhibitor PF-03084014 (Pfizer Global Research and Development) (0.1–0.25 μM), and the combination of the two drugs were administrated 1–2 times at the indicated dosages. Sorafenib resistant cells were generated by exposing 97H spheroids or 97L monolayer cells to sorafenib at concentrations ranging from 5 to 15 μM. The resistant cells were maintained in a low dose of sorafenib (1.25 μM).

### siRNA transfection

97L or 97H cells were transfected with 20 nM of either siRNA-control or siRNA-SNAI1 (Santa Cruz) using Lipofectamine 3000 (Life Technologies) followed by treatment with 1, 3, or 5 μM, respectively, of sorafenib for 48 h. The cells were then seeded into ultra-low attachment plates and cultured in spheroid medium for spheroid colony formation.

### Orthotopic tumor model and drug administration

97H spheroid-derived cancer cells (≥5 × 10^5^) were subcutaneously injected into NOD-severe combined immunodeficiency (SCID) mice to form tumors. The 1–2 mm^3^ tumor cubes were then implanted into the left liver lobes of nude mice as described previously [22]. The mice were randomized to vehicle and 3 treatment groups: (i) vehicle; (ii) PF-03084014 at 100 mg/kg/per day, dissolved in 0.05% methyl cellulose, orally administrated with a 7-day-on/7-day-off schedule; (iii) sorafenib at 30 mg/kg/per day, in the same schedule as PF-03084014; (iii) PF-03084014 + sorafenib at the same dose as single drug treatment, with separate administration at 10:00 AM and 5:00 PM, respectively. The size of each tumor was monitored based on its luciferin (150 mg/kg, I.P. injection) (Gold Biotechnology, MO) signal in an IVIS Spectrum *in vivo* imaging system (PerkinElmer, MA). The size and weight of each tumor was measured at the end point of study. Tumor size was calculated using the formula: tumor volume V = (L × W × W)/2, where L is the length of the tumor and W is the width of the tumor. The tumor tissues were also frozen or fixed for further analysis. Mouse body weight was measured daily. All mouse experiments were approved by the Committee on the Use of Live Animals of the University of Hong Kong (CULATR 4410-17).

### qRT-PCR

Total RNA was extracted using an RNeasy kit (Qiagen). cDNA was produced using a high capacity cDNA reverse transcription kit (Life Technologies). Quantitative PCR was performed in duplicate using the Selected SYBR Green master mix (Life Technologies) on an ABI 7900HT Detection System. The PCR primers are listed in supporting information Supplementary Table 1. Gene expression was quantified based on the CT value and normalized to the levels of 18S.

### Western blots

Polyvinylidene difluoride membranes containing electrophoretically separated proteins from whole-cell lysates and tumor tissues were probed with antibodies against Snail1, E-cadherin (Santa Cruz Biotechnology), phospho-Akt (S473), phospho-Erk1/2 (T202/T204), Nanog, Oct4, Sox2 (all from Cell Signaling Technology), and β-actin (Sigma-Aldrich). The resultant immune complexes were visualized using enhanced chemiluminescence detection reagents (Bio-Rad).

### Immunohistochemistry

Deparaffinized tumor sections were boiled in a microwave for 10 min in citrate buffer for antigen unmasking. After blocking with 1% H_2_O_2_ and 10% goat serum, the sections were incubated with Ki-67 and CD31 (both from Santa Cruz Biotechnology) per the manufacturer’s protocols and visualized using polymer HRP-conjugated secondary antibody (DacoCytomation, Hamburg, Germany). The sections were counterstained with hematoxylin. Sections were examined for positive signals, which were counted from more than 5 random 40× fields. Ki67-positive cells and CD31-positive vessel density was quantified as a percentage.

### Tissue specimens

Tumor tissue specimens were collected from the orthotopic tumors and immediately frozen for tissue lysates or fixed for tissue sections.

### Statistical analysis

The results for variables are presented as the means ± SD. Treatment groups were compared with controls, using independent or paired Student’s *t* test. Pearson Correlation was used for a linear relationship analysis. Genomic gene expression of NOTCH1 and SNAIL1, VIM, and TWIST1 in liver cancers were obtained from the TCGA Liver Cancer database, using UCSC Xena functional genomics explorer (University of California, Santa Cruz). SPSS 21 (IBM Corp.) was applied for all statistical analyses. *P* values < 0.05 were considered statistically significant. To calculate efficacy of combination therapy, synergistic effect is considered when the effect of two drugs in combination is greater than the sum of separate effect of the individual drug; whereas addictive effect is equal to the sum of the effect of the individual drug.

## SUPPLEMENTARY MATERIALS FIGURES AND TABLE



## Abbreviations

GSI: γ-secretase inhibitor
HCC: hepatocellular carcinoma
CSCs: cancer stem cells
EMT: epithelial-mesenchymal transition
97H: MHCC97H
TACE: transarterial chemoembolization
