# Supplementary material for: Synergistic antitumor effect of a γ-secretase inhibitor PF-03084014 and sorafenib in hepatocellular carcinoma
Source: Oncotarget. 2018 Oct 9;9(79):34996–5007. doi: 10.18632/oncotarget.26209 (PMC6201862; doi:10.18632/oncotarget.26209)
Supplement: Supplementary file 1 [file oncotarget-09-34996-s001.pdf]

Synergistic antitumor effect of a γ-secretase inhibitor PF-03084014 and sorafenib in hepatocellular carcinoma

SUPPLEMENTARY MATERIALS

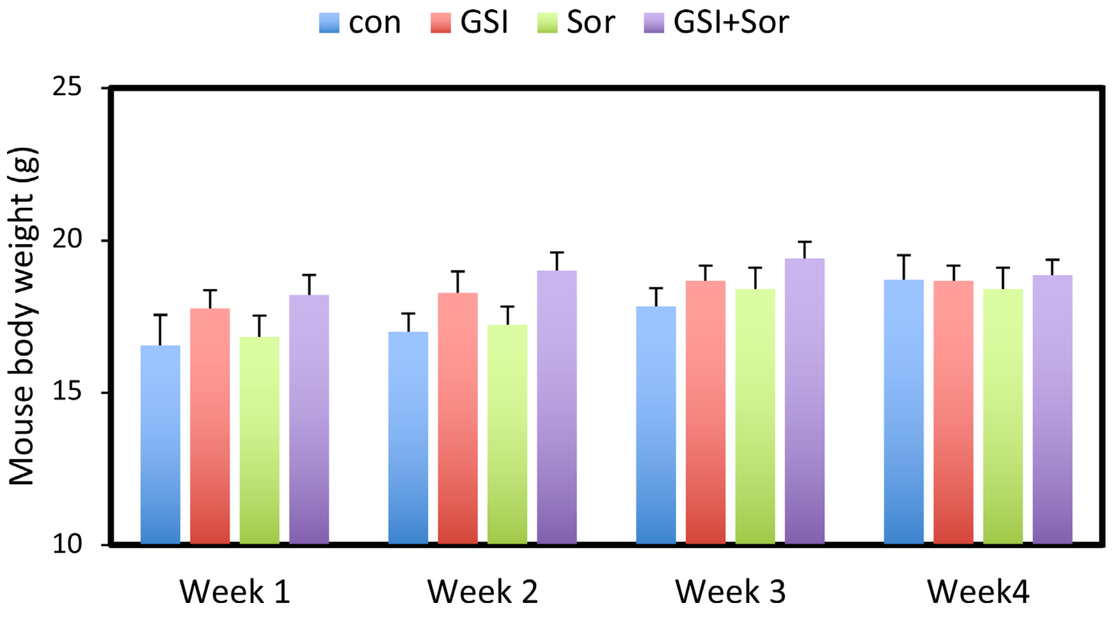

Supplementary Figure 1: In the HCC orthotopic model, mice were treated with vehicle, PF-03084014, sorafenib, or PF-03084014 + sorafenib, respectively, as shown in Figure 2A. Mouse body weight was monitored and recorded daily from weeks 1 to 4. Body weights from each treatment group are presented as the mean ± SD, *n* = 7.

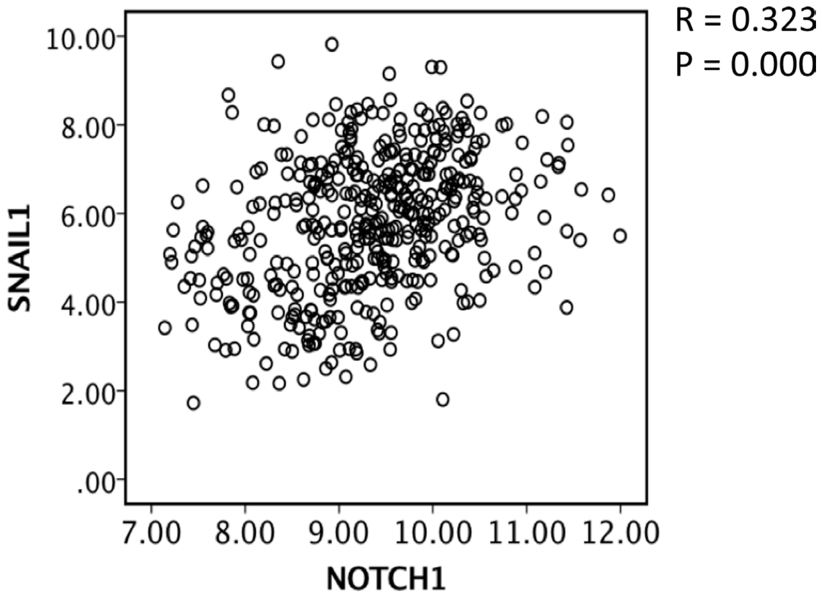

Supplementary Figure 2: Correlation between Notch1 and Snail1 expression levels from 424 liver cancer patients. The data were collected from The Cancer Genome Atlas-Cancer Genome (TCGA liver cancer), and analyzed using SPSS bivariate correlation. Pearson correlation $R = 0.323$, $P = 0.000$ (2-tailed).

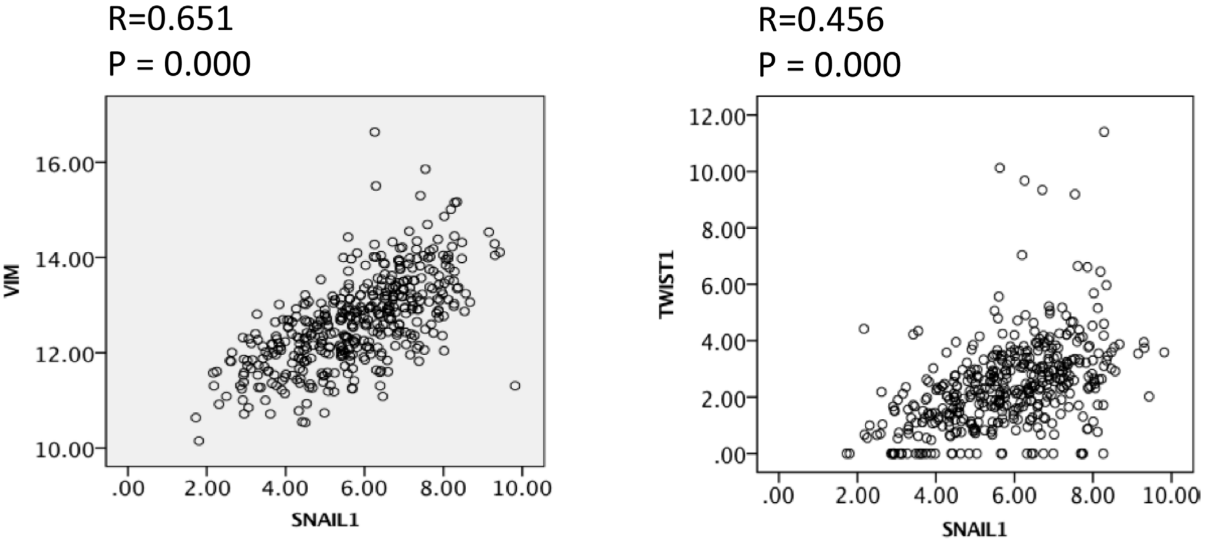

Supplementary Figure 3: Correlation of expression levels between SNAIL1 and VIMENTIN (left panel), and SNAIL1 and TWIST1 (right panel) from 424 liver cancer patients (TCGA liver cancer) and analyzed using SPSS bivariate correlation. Pearson correlation $R = 0.651$, $P = 0.000$ (2-tailed) and $R = 0.456$, $P = 0.000$, respectively.

Supplementary Table 1: Primer sequences

Primer	Sequence (5′ to 3′)<br>F: forward; R: reverse
NOTCH1	F: CAACATCCAGGACAACATGG
	R: GGACTTGCCCAGGTCATCTA
JAG1	F: GACTCATCAGCCGTGTCTCA
	R: TGGGGAACACTCACACTCAA
NANOG	F: CAAAGGCAAACAACCCACTT
	R: TCTGCTGGAGGCTGAGGTAT
OCT4	F: CTCACCCTGGGGGTTCTATT
	R: CTCCAGGTTGCCTCTCACTC
SOX2	F: GCTGCGAACAGTCAGACAGA
	R: ACCTCCCGTCCAAGGTAGG
KLF4	F: CCCACACAGGTGAGAAACCT
	R: ATGTGTAAGGCGAGGTGGTC
SNAIL1	R: CACTATGCCGCGCTCTTTC
	F: GGTCGTAGGGCTGCTGGAA
SNAIL2	F: GAGCATTTGCAGACAGGTCA
	R: GCTTCGGAGTGAAGAAATGC
CDH1	F: CGACCCAACCCAAGAATCTA
	R: AGGCTGTGCCTTCCTACAGA
CDH2	F: GACAATGCCCCTCAAGTGTT
	R: CCATTAAGCCGAGTGATGGT
ABCG2	F: CACCTTATTGGCCTCAGGAA
	R: CCTGCTTGGAAGGCTCTATG
ABCB1	F: GCCTGGCAGCTGGAAGACAAATAC
	R: ATGGCCAAAATCACAAGGGTTAGC
